# Editorial: Management of Intangible Assets Among Non-profit Organizations: Challenges and Peculiarities

**DOI:** 10.3389/fpsyg.2021.771640

**Published:** 2021-10-29

**Authors:** Paula Benevene, Barbara Barbieri, Michela Cortini, Maria Luisa Farnese, Eric Kong, Maria L. Vecina

**Affiliations:** ^1^Department of Human Sciences, Libera Universitá Maria SS. Assunta, Rome, Italy; ^2^Department of Political and Social Sciences, University of Cagliari, Cagliari, Italy; ^3^Department of Psichological Sciences, Health and Territory, University of Studies G. d'Annunzio Chieti and Pescara, Chieti, Italy; ^4^Department of Psychology, Sapienza University of Rome, Rome, Italy; ^5^School of Management and Enterprise, University of Southern Queensland, Toowoomba, QLD, Australia; ^6^Departamento de Psicología Social, del Trabajo y Diferencial, Complutense University of Madrid, Madrid, Spain

**Keywords:** intangible assets, non-profit organizations, third sector, non-profit management, knowledge structures and processes

Non-profit organization (NPO) is a broad term that includes different types of entities, such as associations, foundations, voluntary organizations, charities, and social advocacy groups, as well as different kinds of structures that include hospitals, universities, and trade organizations. They sustain the active participation of citizens with the aim of improving the quality of life of individuals and their communities. Their actions span from funding research to advocacy, and from social services to the protection of historical heritage, yet all of them mainly offering services that are intangible by their own nature.

Notwithstanding the variety of forms and activities they perform, all NPOs share similar features. Indeed, NPOs are usually formally structured, private and do not depend on the state, even though they might receive funds from local or national governments for their activities. They are non-profits, meaning that they operate for purposes other than generating profit and they do not distribute profits to their directors, managers and stakeholders. Moreover, NPOs are usually self-governed, with their own mechanisms for internal governance, and they rely on some degree of voluntary work.

These structural features are strictly interwoven with management issues such as the need to balance business and social aims (explicitly aiming to benefit the community; Evers, 2005). and to complement limited financial and tangible resources with intangible assets, such as volunteering (Laville and Nyssens, 2001) and the enhancement of their members' active participation, loyalty and commitment (Benevene et al., 2017).

According to Drucker (1993), knowledge is the only source of sustainable competitive advantage and it has proven to play a key role in achieving excellence and innovation in NPOs and beyond, generating a positive impact on their present as well as on their future performance. Knowledge adds value to organizations through the management and development of intangible assets, such as positive relationships with customers and end-users, a know-how not easily imitable by competing organizations, higher organization's reputation, volunteers' and employees' commitment, and donors' loyalty. It is for this reason that Sveiby (2001) has defined intangible assets as knowledge structures.

Overall, the key value of their employees' knowledge and values, and the pivotal role of the professional–citizens relationship in pursuing their mission, make intangible resources a crucial asset for these organizations, if not the only resources NPOs can rely on.

Furthermore, in the past two decades these organizations have been experiencing an ever-growing demand to provide services, due to the rise of economic and social problems, as well as the reduction of services funded by local and national governments. At the same time, they are also facing a substantial reduction in public funds for performing their activities. Moreover, NPOs are confronted with increasing competition among themselves for volunteers, donors, and resources. The survival and growth of these organizations in an increasingly competitive environment depends even more on their ability to manage and develop their knowledge.

Building on these considerations, this Research Topic aimed to deepen the current knowledge on the role of intangible assets in NPOs from interdisciplinary psychological perspectives.

The papers collected in this special issue cover a wide range of areas and topics, as well as geographical areas. For instance, while most of these studies were developed among Western countries, where NPOs have a long history and a more consolidated background, a relevant contribution is offered from Poland and China, where NPOs have grown tremendously over the past two decades. In fact, the article of Li and Zhang highlights how collectivism, a prominent value in eastern cultures, plays a moderating role between volunteers' motivation and their organizational identity, which is a pivotal factor in the retaining volunteers. From a management point of view, the findings of this study suggest that NPOs should pay attention to recruiting candidates with a high level of altruism and collectivism and train them to hold management positions in the future.

A second study by Kanafa-Chmielewska deals with volunteers' personality traits, comparing volunteers engaged in different activities, namely: politics, religious communities, and humanitarian aid. Their results suggest that volunteers show different personality traits, according to the NPOs they are serving (e.g., those engaged in politics show higher levels of pragmatism).

Other studies approached the management of intangibles among NPOs in terms of organizations' performance. More specifically, Moreno-Albarracín et al. developed a management model, based on the Spanish National Organization for the Blind (ONCE), that aims at assessing the management of social services run by NPOs, identifying a set of indicators where efficiency, effectiveness, and excellence are key criteria.

Similarly, Treinta et al. present an exhaustive literature review of performance measurement among NPOs, showing how this issue is still underdeveloped. Their findings highlight how measuring the performance of NPOs is far more complex than it is for for-profit companies, since NPOs are value-oriented, socially engaged, and must deal with many different stakeholders.

The article by Buonomo et al. reviewed the effect of intangible assets, namely intellectual capital, on NPOs' performance. Their results show that all the three dimensions of intellectual capital (human, organizational, and relational capital) influence NPOs' performance, though human capital seems to be considered the most powerful to generate organizational growth.

Ripamonti et al. tackled the intellectual capital of NPO from the perspective of a group of managers of a specific kind of NPOs, namely the trade unions. This qualitative study highlights four different ways of conceiving the role of people in the organization that, in turn, can make a big difference to the management of intangible assets.

Another study of Benevene et al. addressed the impact of leadership on volunteers, showing that leaders' actions oriented toward the enablement of learning and innovation may influence volunteers' affective commitment, through the full mediation of volunteer satisfaction.

The perspective of volunteers was addressed in the article of Giancaspro and Manuti, who interviewed a group of young volunteers. This study reveals how voluntary work proves to be a context of informal and non-formal learning and vocational guidance, useful to develop skills and abilities that may increase volunteers' future employability.

Finally, an innovative study carried out by Zito et al. observed the role played by brand, culture, and reputation as intangible goods in creating a link between donors and NPOs. In fact, they assessed the effectiveness of the Unicef bequest campaign in terms of emotional response, applying neuromarketing tools such as eye-tracking.

Undoubtedly, the studies collected in this Research Topic are far from being exhaustive of all the issues raised by the management of intangible assets among NPOs. However, they can offer a variety of hints on the possible future directions, challenges, opportunities, and peculiarities of these organizations.

## Author Contributions

All authors contributes equally on the paper planning, developing, and writing.

## Conflict of Interest

The authors declare that the research was conducted in the absence of any commercial or financial relationships that could be construed as a potential conflict of interest.

## Publisher's Note

All claims expressed in this article are solely those of the authors and do not necessarily represent those of their affiliated organizations, or those of the publisher, the editors and the reviewers. Any product that may be evaluated in this article, or claim that may be made by its manufacturer, is not guaranteed or endorsed by the publisher.
